# Cholesterol-modified Hydroxychloroquine-loaded Nanocarriers in Bleomycin-induced Pulmonary Fibrosis

**DOI:** 10.1038/s41598-017-11450-3

**Published:** 2017-09-06

**Authors:** Li Liu, Jun Ren, Zhiyao He, Ke Men, Ye Mao, Tinghong Ye, Hua Chen, Ling Li, Bocheng Xu, Yuquan Wei, Xiawei Wei

**Affiliations:** State Key Laboratory of Biotherapy/Collaborative Innovation Centre, Sichuan University, No. 17, Block 3, Southern Renmin Road, Chengdu, Sichuan 610041 China

## Abstract

An increasing number of reports have suggested the use of hydroxychloroquine (HCQ) as an adjunct anti-cancer treatment to enhance the chemotherapeutic response, as well as for the treatment of several fibrotic skin diseases and cystic fibrosis. In this study, we synthesized a cholesterol-modified HCQ (Chol-HCQ) and hypothesized that a systemic delivery system with Chol-HCQ nanocarriers could be effective for the treatment of bleomycin-induced pulmonary fibrosis. Chol-HCQ significantly inhibits the proliferation of rat lung fibroblasts, regulates inflammation and ameliorates bleomycin-induced pulmonary fibrosis in rats. It regulates the expression of pro-inflammatory cytokines, such as TNF-α; reduces the infiltration of inflammatory neutrophils; and inhibits the phosphorylation of NF-κB. Chol-HCQ also reduces the expression of connective tissue growth factor (CTGF) and phosphorylation of extracellular regulated protein kinase (p-ERK) in rats with bleomycin-induced pulmonary fibrosis. Chol-HCQ nanocarriers reduce early pulmonary inflammation and inhibit the CTGF/ERK signalling pathway in bleomycin-induced pulmonary fibrosis. These results demonstrate that Chol-HCQ liposomes suppress pulmonary inflammation and reduce pulmonary fibrosis induced by bleomycin. The systemic administration safety of Chol-HCQ liposomes was confirmed after intravenous administration for 28 days in rats. The present study provides evidence that Chol-HCQ liposomes may be a potential therapeutic agent for inflammation associated with pulmonary fibrosis.

## Introduction

Idiopathic pulmonary fibrosis (IPF) is a disease characterised by alveolar epithelial cell injury and hyperplasia, inflammatory cell accumulation, and fibroblasts hyperplasia^1^ that results in the deposition of extracellular matrix, including fibrillar collagens, fibronectin, elastic fibres, and proteoglycans^2^. As previously reported, patients with IPF have a mean survival of only 2–4 years^3^. Numerous clinical trials on research for effective treatment agents have been conducted^4^. For these reasons, novel therapeutic agents are strongly needed. Antimalarial chloroquine(CQ) and hydroxychloroquine (HCQ) have been proven to exert anti-inflammatory effects through down-regulation of pro-inflammatory cytokines, such as tumour necrosis factor alpha (TNF-α) and interlukin1β (IL-1β)^5, 6^. It was also reported that HCQ effectively inhibited the proliferation of fibroblasts, suppressed metabolic activities in fibrotic skin diseases and inhibited extracellular signalling-regulated kinase ERK1/2 activation in human dermal fibroblasts^7^. Proliferation of lung fibroblasts represents a key source of interstitial collagens, and the hallmark lesions are the fibroblastic foci that represent focal areas of active fibrogenesis and feature vigorous fibroblast replication and exuberant extra-cellular matrix deposition, which may lead to obliteration of the distal air space^8^. Connective tissue growth factor (CTGF) has been shown to modulate many signalling pathways that lead to tissue remodelling and fibrosis, including cell adhesion and migration, angiogenesis, myofibroblast activation, and extracellular matrix deposition and remodelling^9^. It has been considered as a prognostic marker in fibrotic diseases and a potential candidate in new anti-fibrotic therapy approaches^10^.

Cholesterol, which is known as one of the most common endogenous physiological molecules, plays an important role in the self-assembly of lipopolymer molecules in biological environments^11, 12^. Cholesterol modification is wildly used as a membrane-anchoring strategy^13^; for example, Hedgehog proteins membrane mediate anchoring with this lipophilic modification^13, 14^ as inhibitors that are localized to raft domains, which are enriched for β secretase. The modified strategies involving cholesterol have been designed and exploited for their potential to enhance the half-life and antiviral activity of peptides, which is based on a cholesterol tag at the N-terminus of the fusion-inhibitory peptide^15^. Based on these hypothesises, cholesterol-modified HCQ might show better characteristics. HCQ has been reported to effectively induced apoptosis at 1–10 μM concentrations in human dermal fibroblasts^7^. Cholesterol-modified HCQ showed a lower toxicity to lung fibroblasts and inhibited proliferation at 10 μM, but induced apoptosis at a higher concentration (50 μM) compared to HCQ. More studies are needed to uncover the mechanism.

The size of the nanocarriers those we prepared are typically small (from a few tenths to a few hundreds of nanometres) to insure systemic (intravenous) administration. Neutrophils are responsible for damage for tissue as a result of their capacity to release reactive oxygen species (ROS), superoxide and a number of destructive proteases alongside inflammatory cytokines^16^. In this study, we investigated the immune regulatory functions of Chol-HCQ liposomes in bleomycin-induced pulmonary fibrosis in rats. Here, we show that Chol-HCQ inhibited the proliferation of lung fibroblasts and regulated pulmonary inflammation induced by bleomycin by suppressing the expression of pro-inflammatory cytokines, such as TNF-α, reduced infiltration of inflammatory neutrophils and inhibited the phosphorylation of NF-κB and ERK. The inhibition of ERK and NF-κB might contribute to the resolution of early inflammation in the pulmonary fibrosis development. Chol-HCQ liposomes reduced early pulmonary inflammation and inhibited the CTGF/ERK signalling pathway to help against bleomycin-induced pulmonary fibrosis. The anti-fibrotic effects of Chol-HCQ liposomes were better than HCQ liposomes. These results demonstrated that Chol-HCQ liposomes could prevent bleomycin-induced pulmonary fibrosis and might be a novel anti-fibrotic agent for the treatment of inflammation associated with pulmonary fibrosis.

## Results

### Chol-HCQ suppresses lung fibroblast proliferation and inhibits ERK1/2 and NF-κB phosphorylation

**Figure 1:**
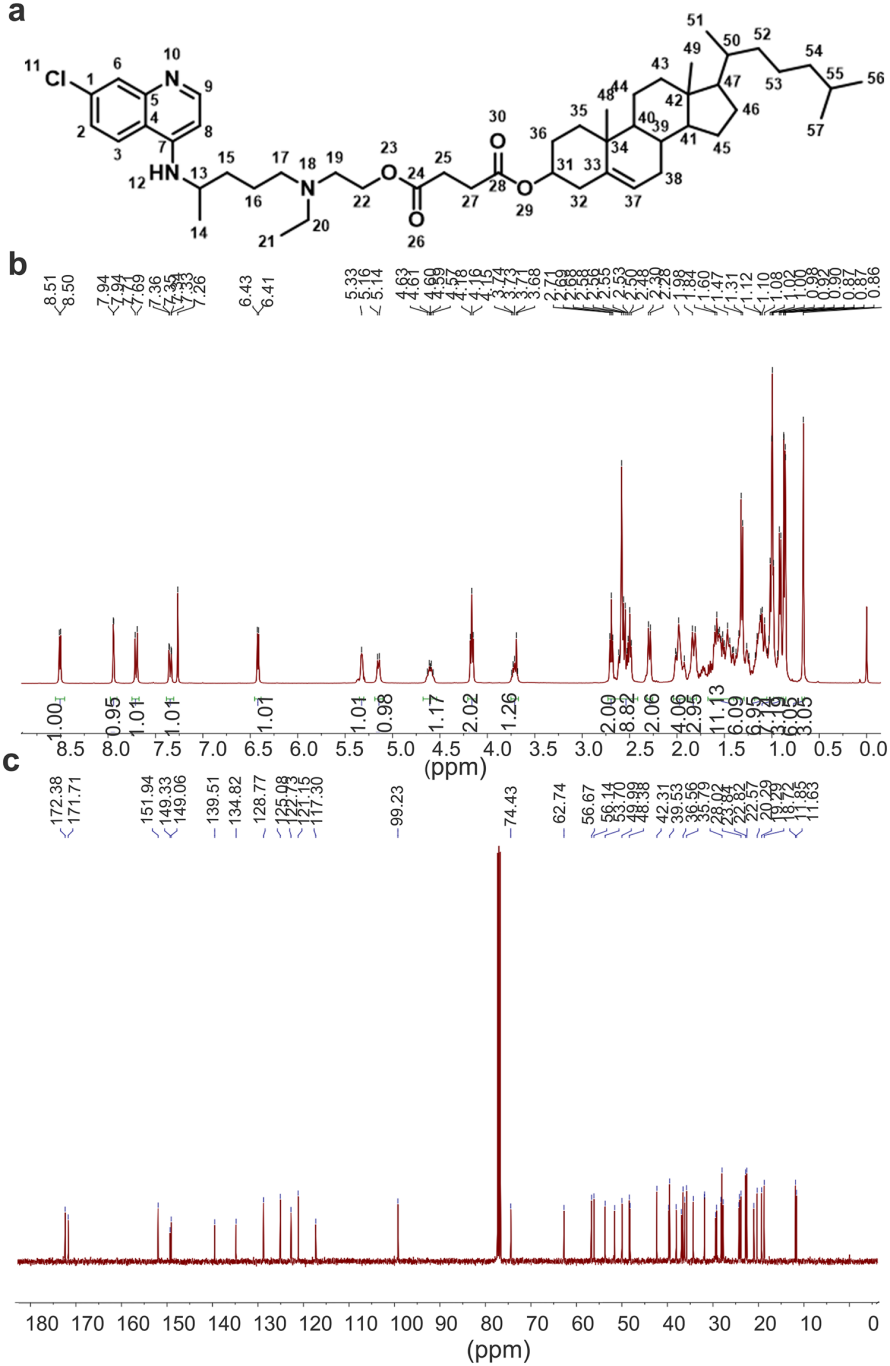
Chol-HCQ characterization. (**a**) The chemical structural of Chol-HCQ. (**b**) The Chol-HCQ 1H-NMR spectrum. ^1^H-NMR (400 MHz, CDCl_3_): δ = 8.51 (d, *J* = 5.4 Hz, 1 H, 9), 7.94 (d, *J* = 1.8 Hz, 1 H, 6), 7.70 (d, *J* = 9.0 Hz, 1 H, 3), 7.34 (dd, *J* = 8.9, 1.9 Hz, 1 H, 2), 6.42 (d, *J* = 5.5 Hz, 1 H, 8), 5.33 (s, 1 H, 12), 5.15 (d, *J* = 7.2 Hz, 1 H, 37), 4.68 − 4.54 (m, 1 H, 31), 4.16 (t, *J* = 6.1 Hz, 2 H, 22), 3.75 − 3.67 (m, 1 H, 13), 2.69 (t, *J* = 6.1 Hz, 2 H, 19), 2.66 − 2.41 (m, 9 H, 13, 17, 20, 25, 27), 2.29 (d, *J* = 7.9 Hz, 2 H, 32), 2.04 − 1.91 (m, 4 H), 1.86 − 1.79 (m, 3 H), 1.67 − 1.40 (m, 11 H), 1.37−1.29 (m, 6 H), 1.18 − 1.05 (m, 7 H), 1.02 − 0.98 (m, 7 H), 0.91 (d, *J* = 6.5 Hz, 3 H, 51), 0.86 (dd, *J* = 6.5, 1.2 Hz, 6 H, 56, 57), 0.67 (s, 3 H, 49). (**c**) The Chol-HCQ 13C-NMR spectrum. ^13^C-NMR (101 MHz, CDCl_3_): δ = 172.38 (24), 171.71 (28), 151.94 (9), 149.33 (5), 149.06 (7), 139.51 (33), 134.82 (1), 128.77 (6), 125.08 (3), 122.73 (37), 121.15 (2), 117.30 (4), 99.23 (8), 74.43 (31), 62.74 (22), 56.67 (17), 56.14 (41), 53.70 (47), 51.60 (19), 49.99 (13), 48.37 (40), 48.21 (20), 42.31 (42), 39.72 (54), 39.52 (43), 38.07 (32), 36.93 (35), 36.56 (34), 36.19 (52), 35.79 (50), 34.31 (38), 31.88 (39), 31.83 (25), 29.41 (27), 29.20 (36), 28.23 (55), 28.02 (46), 27.75 (45), 24.28 (53), 24.15 (15), 23.84 (16), 22.82 (57), 22.57 (56), 21.02 (14), 20.29 (44), 19.29 (51), 18.72 (48), 11.85 (21), 11.63 (49).

**Figure 2:**
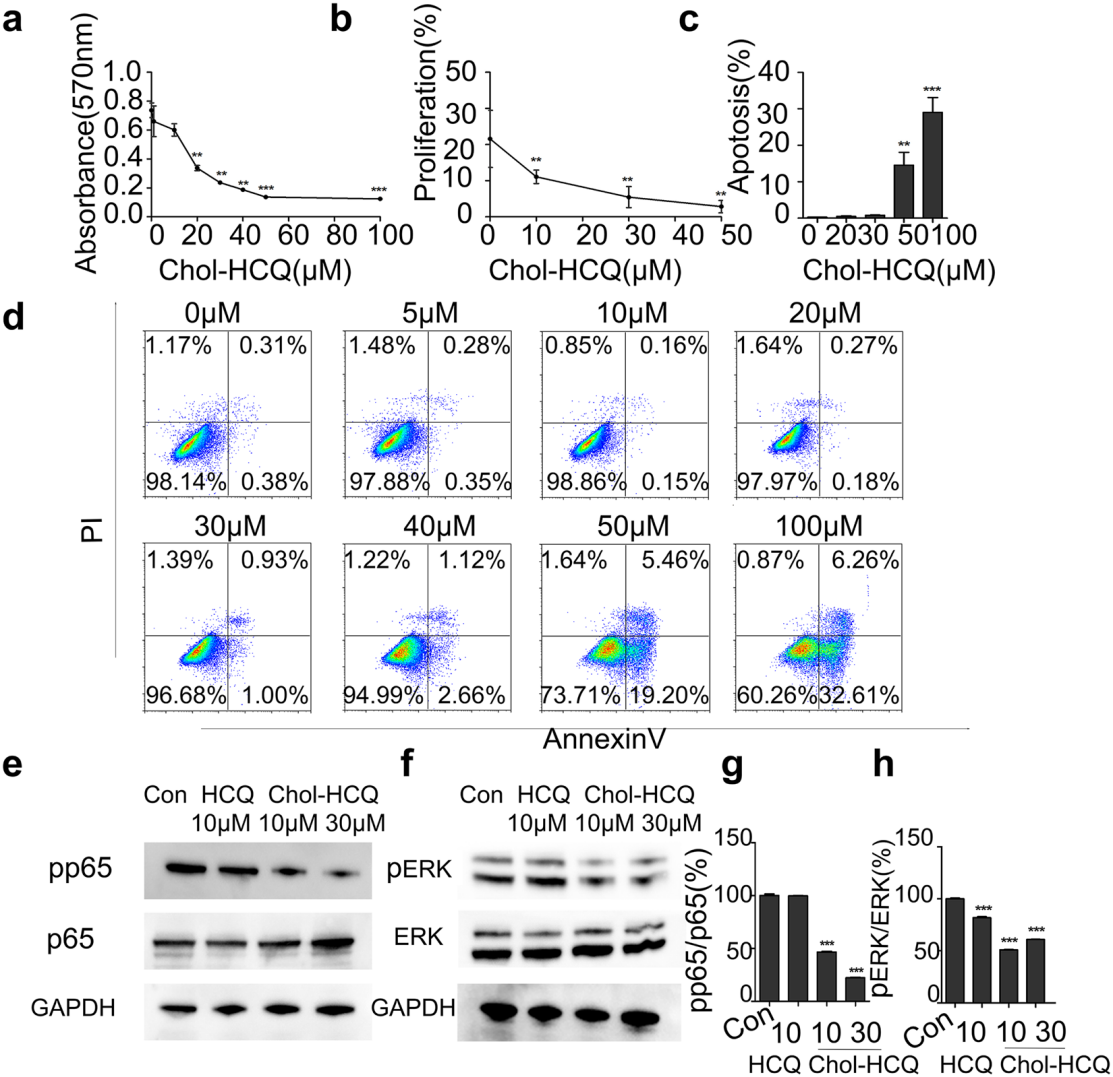
Chol-HCQ inhibits the lung fibroblast proliferation via NF-κB and ERK pathways. The lung fibroblasts were obtained from bleomycin-treated rats as described and the cells were seeded in a 96-well plate. The cells were treated with Chol-HCQ (0-100μΜ) for 24 hours and tested by (**a**) MTT or (**b**) by the percentages of EdU-stained lung fibroblasts. (**c** and **d**) For the apoptosis analysis, lung fibroblasts were treated with Chol-HCQ for 48 hours followed by Annexin V/PI staining and examined by FACS analysis. Similar results were obtained in three separate experiments. (**e**–**h**) Chol-HCQ inhibits ERK1/2 and NF-κB phosphorylation in lung fibroblasts. Chol-HCQ decreases ERK1/2 phosphorylation dose-dependently at Thr202/Tyr204 and NF-κB phosphorylation. The data are representative of three separate experiments. *p < 0.05, **p < 0.01, and ***p < 0.001.

The general procedure for the synthesis of Chol-HCQ is shown in Supplementary Figure 1a. The synthesis of the novel compound is fully described in the Methods. The product was used for administration after further characterization by ^1^HNMR spectroscopy (Fig. 1b), ^13^C-NMR spectroscopy (Fig. 1c) and a purity assay using high-performance liquid chromatography (data not shown). Chol-HCQ inhibits the proliferation of lung fibroblasts isolated from bleomycin-treated rats in a dose-dependent manner (Fig. 2a). The results were confirmed directly by calculating the EdU immunofluorescent-stained cells (Fig. 2b). Lung fibroblasts were treated with different concentrations of Chol-HCQ (0 to 100 μM) for 48 hours and the cell apoptosis was analysed by flow cytometry analysis after Annexin V/PI staining as described in the Methods. We found that Chol-HCQ inhibited lung fibroblast proliferation from low concentrations (10 μM) and induced apoptosis at higher concentrations (50 to 100 μM) (Fig. 2c and d). HCQ was reported to induce skin fibroblast apoptosis at lower concentrations (10–20 μM)^7^, and Chol-HCQ showed lower toxicity than HCQ. Chol-HCQ inhibited NF-κB phosphorylation in the lung fibroblasts (Fig. 2e and g). Chol-HCQ also significantly decreased ERK1/2 phosphorylation at Thr202/Tyr204 (Fig. 2f and h).

### Administration of Chol-HCQ liposomes decreases bleomycin-induced pulmonary fibrosis in rats

**Figure 3:**
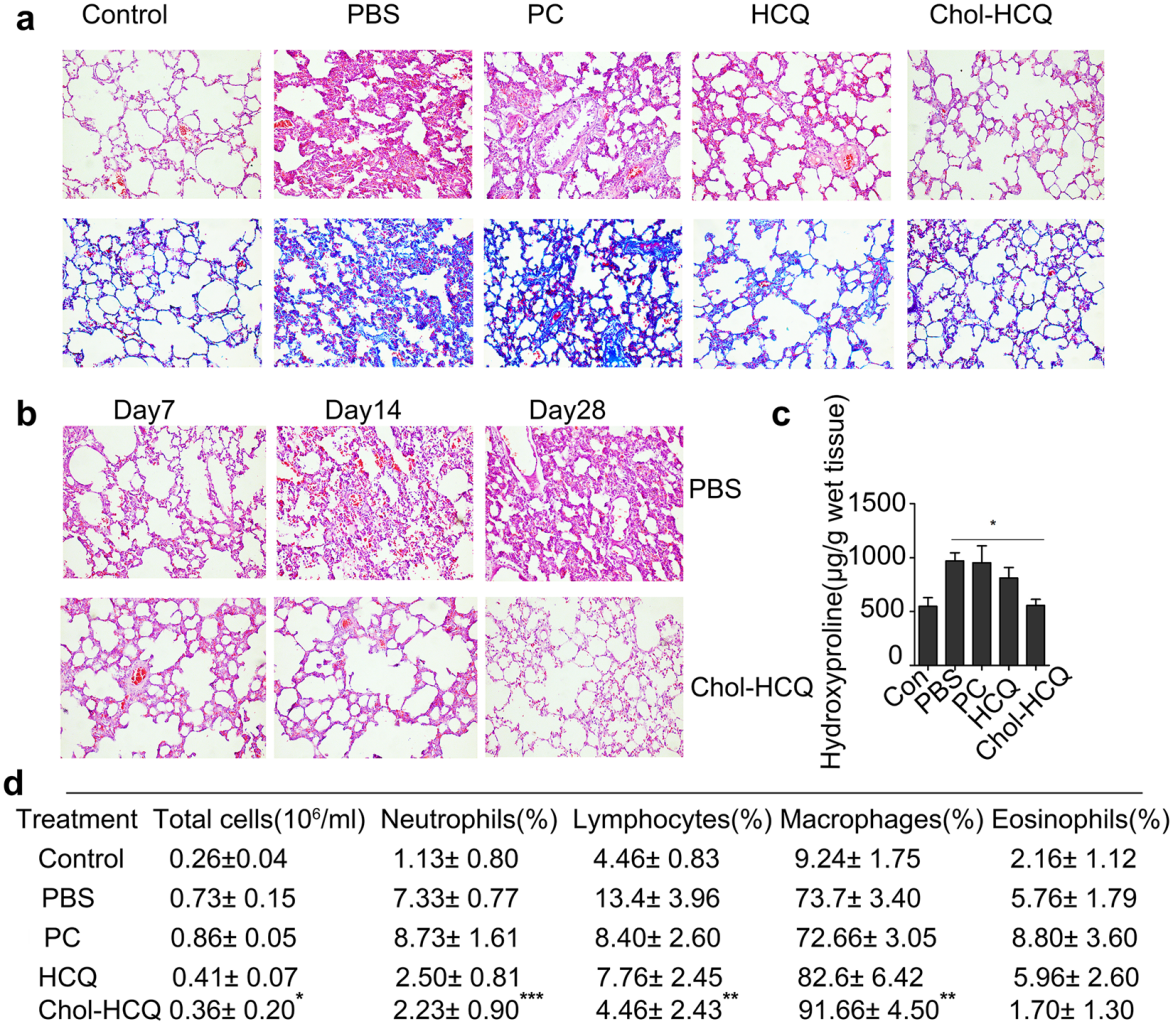
Histological examinations of the anti-fibrotic effects and the time kinetic of Chol-HCQ liposomes in bleomycin-induced pulmonary fibrosis. Rats were treated with bleomycin and then injected with Chol-HCQ liposomes (20 mg/kg/day) and HCQ liposomes (8 mg/kg/day) via the tail vein; PBS solution and null liposomes (PC) were used as controls. (**a**) On day 28, the rats were sacrificed and histological examination was performed by H&E staining (up) and Masson’s trichrome staining (below). Original magnification is 200x (n = 6); the data are representative of three separate experiments. (**b**) Bleomycin-induced rats were treated with Chol-HCQ liposomes or PBS as a control. On days 7, 14 and 28, the rats were sacrificed and histological examination was performed by H&E staining (n = 4). (**c**) The hydroxyproline contents in the lung tissues were examined on day 28 (n = 6). (**d**) Lung lavages were collected and examined by differential cell counting in the BALF on day 28 of the experiment; the total cell numbers, neutrophils, macrophages, eosinophils and lymphocytes were counted. Original magnification is 200x; data are representative of three separate experiments. *p < 0.05.

We further studied the *in vivo* anti-fibrotic effects of Chol-HCQ in a bleomycin-induced pulmonary fibrosis model in SD rats treated as described in the Methods. On day 28 of the experiment, the experimental rats were sacrificed and their lungs were harvested. The H&E staining sections were made as described in the Methods. The bleomycin-treated rats were injected intravenously with Chol-HCQ, CQ liposomes, PBS solution and null PC liposomes as a control. A reduction in fibrotic lesions was observed in the H&E staining sections from Chol-HCQ and HCQ liposome-treated rats (Fig. 3a, up). Masson’s trichrome staining (Fig. 3a, below) confirmed the results. In fact, study on the time kinetics of bleomycin-induced pulmonary fibrosis was conducted after bleomycin treatment; the rats were sacrificed on days 7, 14 and 28 of the experiment. We found that Chol-HCQ significantly inhibited bleomycin-induced pulmonary fibrosis from the early stage (Day 14) (Fig. 3b). However, we clearly observed that Chol-HCQ inhibited lung fibroblast proliferation *in vitro* (Fig. 2a and b). Altogether, we inferred that Chol-HCQ might effectively suppress pulmonary fibrosis via regulating the inflammation induced by bleomycin at the early fibrotic stage.

We also examined the hydroxyproline content in the lung tissues of bleomycin-treated rats (Fig. 3c). Chol-HCQ liposome treatment markedly reduced the levels of hydroxyproline compared to the control groups, and the anti-fibrotic effects of the Chol-HCQ liposomes was better than the HCQ liposomes; these results were highly consistent with the Masson’s trichrome data (Fig. 3a, below). We collected lung lavage and performed differential cell counting on the bronchoalveolar lavage (BALF) on day 28 of the experiment (Fig. 3d). There was a reduction in neutrophils, lymphocytes and the total cell numbers in Chol-HCQ-treated rats compared to PBS-treated; macrophages increased in Chol-HCQ-treated rats, the numbers of eosinophils were unaltered.

### Chol-HCQ liposomes regulate pulmonary inflammation induced by bleomycin and prevent pulmonary fibrosis by inhibiting the CTGF/ERK signalling pathway

**Figure 4:**
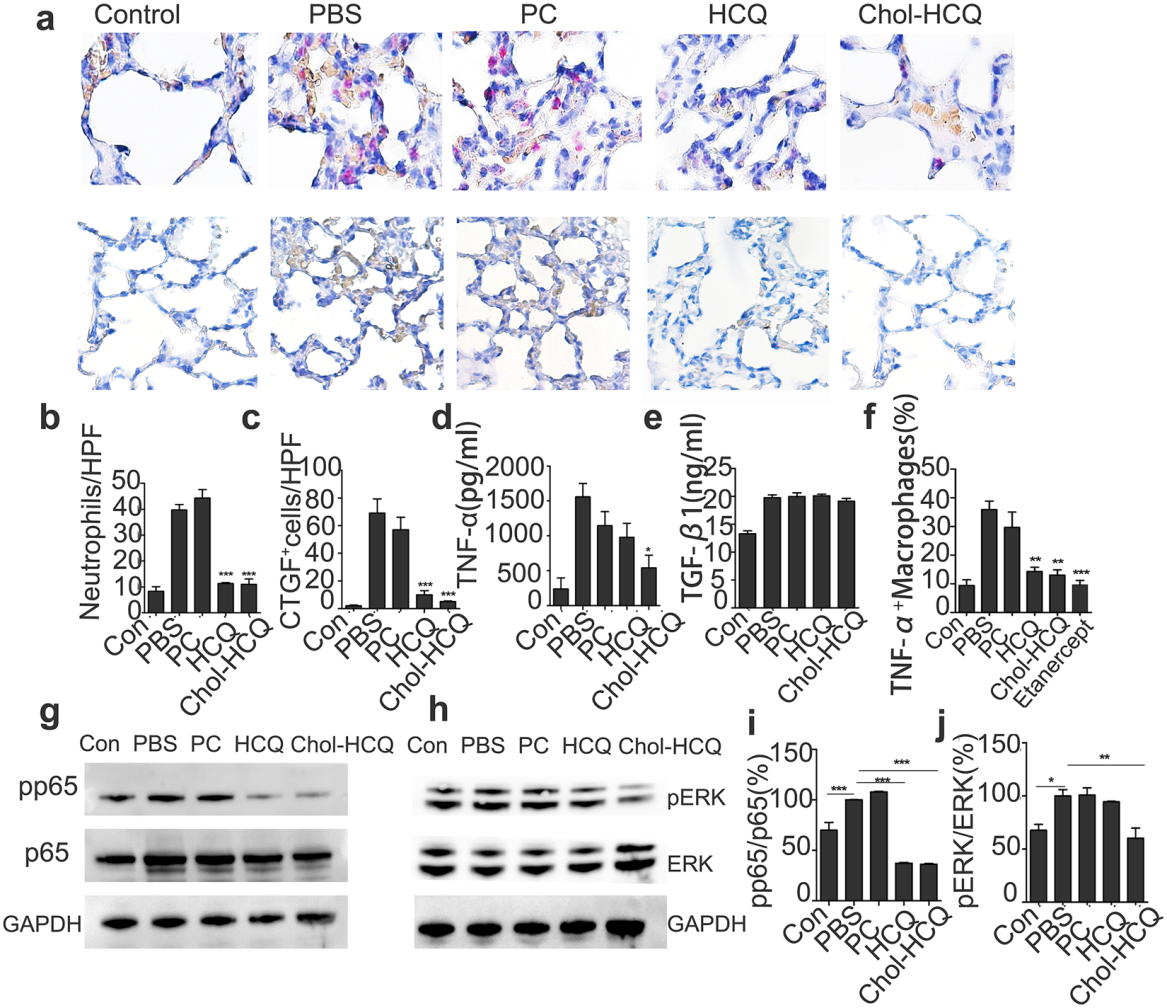
Chol-HCQ liposomes suppress bleomycin-induced pulmonary fibrosis through anti-inflammatory effects and by inhibiting the CTGF/ERK signalling pathways. (**a**, up) Specific esterase staining of neutrophils in rat lung sections on day 7 of the experiment. The rats were treated with bleomycin before administration (400×). (**b**) Neutrophils were counted in 5 random 200 s fields. (**a**, below, and **c**)Immunohistochemistry analysis of CTGF levels in the lung tissues from BLM-induced pulmonary fibrosis rats. Original magnification is 200 s. Positive cells were countered in 5 random fields. (**d** and **e**) TNF-α and TGF-β1 contents in plasma from rats on day 7 were determined by ELISA kits. (f) Isolated alveolar macrophage cells were stimulated with bleomycin (25 μg/ml) and treated with Chol-HCQ (10 μM) or HCQ (10 μM) and etanercept (5 μg/ml) in the presence of brefeldin A in 24-well plate at 37 °C for 24 h. Flow cytometry was conducted to detect macrophage intracellular TNF-α expression. (**g**–**j**) On day 14, rats were sacrificed and lung tissues lysate were made for Western blot analysis. Chol-HCQ inhibits ERK1/2 (Thy202/Thr204) and NF-κB phosphorylation *in vivo*. Data are representative of three separate experiments. *p < 0.05, **p < 0.01, and ***p < 0.001.

Pulmonary inflammation induced by bleomycin is characterized by neutrophil infiltration in the lung (Fig. 4a, up, and b). Chol-HCQ and HCQ liposomes dramatically eliminated inflammatory neutrophils in lung tissues, which was highly consistent with the bronchoalveolar lavage assay data (Fig. 3d). Connective tissue growth factor (CTGF) is regarded as one of the key fibrotic cytokines in the development of pulmonary fibrosis. To analyse the anti-fibrotic effects of Chol-HCQ, we examined CTGF expression in lung tissues from the experimental rats by immunohistochemistry staining (Fig. 4a, below, and c). The anti-fibrotic effects of the Chol-HCQ liposomes were confirmed by inhibiting the expression of CTGF.

Studies have suggested that patients with IPF have high levels of TNF-α^17–21^, and TNF-α overexpression has promoted the development of highly progressive pulmonary fibrosis^22^. Chol-HCQ liposomes inhibited TNF-α levels in plasma from bleomycin-induced rats (Fig. 4d). The anti-inflammatory effects of Chol-HCQ liposomes were better than HCQ liposomes. TGF-β1 has both anti-inflammatory and pro-fibrotic activities, which is one of the key drivers of fibrosis^23^. Chol-HCQ and HCQ liposomes have no influence on TGF-β1 levels in plasma in this pulmonary fibrosis model (Fig. 4e). Macrophages isolated from bronchoalveolar lavage were cultured with bleomycin stimulation and treated with Chol-HCQ or HCQ; the TNF-α inhibitor etanercept was used as control, as it is thought be effective at reducing clinically progressive IPF^21^. We found that Chol-HCQ significantly inhibited the expression of TNF-α in the lung macrophages stimulated with bleomycin (Fig. 4f). The activation of NF-κB can induce pro-inflammatory responses. For the study of the inflammation regulatory effects of Chol-HCQ liposomes in pulmonary fibrosis induced by bleomycin, we analysed the phosphorylation of NF-κB in lung tissues from bleomycin-treated rats by Western blot (Fig. 4g and i). Chol-HCQ and HCQ liposomes inhibited the activation of NF-κB in the lung of bleomycin-treated rats. We further investigated the phosphorylation of ERK1/2 (Thr202/Tyr204) by Western blot analysis. The phosphorylation of ERK1/2 decreased in the Chol-HCQ liposome-treated rats compared to the PBS- or PC liposome-treated rats (Fig. 4h and j).

Altogether, these findings indicated that the Chol-HCQ liposomes inhibited the CTGF/ERK signalling pathway, which might contribute to protect the rats against bleomycin-induced pulmonary fibrosis.

### Pharmacokinetics study and safety evaluation of Chol-HCQ liposomes and hydroxychloroquine sulphate

**Figure 5:**
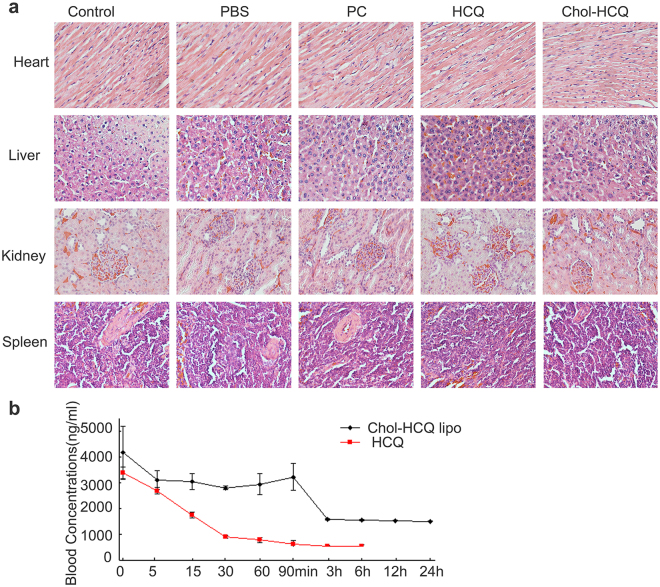
Preliminary safety evaluations of Chol-HCQ liposomes in female rats with bleomycin-induced pulmonary fibrosis. (**a**) Representative H&E images (400x) of vital organs including the heart, liver, spleen and kidney. Chol-HCQ and HCQ liposomes had no obvious toxicity on these tissues. (**b**) The concentrations of Chol-HCQ and HCQ in rat whole blood from 0 to 24 hours after intravenous administration of Chol-HCQ liposomes (total 20 mg/kg, Chol-HCQ 10 mg/kg) and HCQ sulphate (10 mg/kg) were assayed using high performance liquid chromatography (HPLC) (n = 5).

HCQ was reported to have retinal toxicity. Under daily consumption of 4.0 to 5.0 mg/kg, the prevalence of retinal toxicity remained under 1% in the first 10 years and under 4% after 20 years of hydroxychloroquine therapy^24, 25^. Additional major risk factors include renal disease, as the drug is predominately excreted by the kidneys. To study the safe administration of Chol-HCQ liposomes in the experimental rats, representative H&E images (400×) of vital organs including the heart, liver, spleen and kidney were observed. Chol-HCQ and HCQ had no obvious toxicity on these tissues (Fig. 5a). Moreover, blood tests and serological biochemical analyses were performed on day 28. All the biochemical indexes indicated that vital organ functions after Chol-HCQ or HCQ liposome treatment in mice were similar to normal ranges (Supplementary Figure 3). Based on the biochemical indexes and H&E images, it could be inferred that Chol-HCQ liposomes are a relatively safe formulation for intravenous administration for the treatment of female rats treated with bleomycin because the liposomes not enough to induce toxic effects.

To study the pharmacokinetics of Chol-HCQ liposomes and HCQ sulphate, the concentrations in rat whole blood from 0 to 24 hours after intravenous administration were assayed by using high performance liquid chromatography (HPLC) (Fig. 5b). Chol-HCQ concentrations varied between 4272 and 1392 ng/ml after Chol-HCQ liposome administration and HCQ levels ranged between 3415 and 329 ng/ml after HCQ sulphate administration. The encapsulated Chol-HCQ produces high drug levels for a longer time within 24 hours in plasma compared to free HCQ (Fig. 5b).

## Discussion

Our study demonstrates that cholesterol-modified hydroxychloroquine effectively inhibited the growth of lung fibroblasts *in vitro* and suppressed the phosphorylation of NF-κB and ERK1/2 in bleomycin-treated fibroblasts. Furthermore, Chol-HCQ liposomes reduced bleomycin-induced pulmonary fibrosis progression in rats.

HCQ was reported to effectively induce apoptosis at 1–10 μM concentrations in human dermal fibroblasts^7^. Cholesterol-modified HCQ showed lower toxicity to lung fibroblasts; it inhibited proliferation at 10 μM and induced apoptosis at a higher concentration (50 μM) than HCQ. In the administration of liposomal preparations, Chol-HCQ liposomes showed better anti-fibrotic and anti-inflammation effects compared to HCQ liposomes in rats model of pulmonary fibrosis. One of the explanation might be that as cholesterol has been reported as a membrane anchor for hedgehog protein^13^, antifluorescein antibodies^26^ and streptavidin protein^27^ because it is a highly abundant membrane-associated steroid that is functionally linked to endocytosis^28, 29^. Cholesterol derivatives were also used for the delivery of siRNA^30^ and plasmid DNA liposomes for the “anchoring” effect^31^. More researches are needed to confirm the hypothesis that Chol-HCQ own better “anchoring” effect compare to HCQ.

The mechanistic studies revealed that Chol-HCQ inhibited lung fibroblast proliferation and suppressed the phosphorylation of NF-κB and ERK1/2. Chol-HCQ liposomes inhibited TNF-α levels in plasma from bleomycin-induced rats and showed significant elimination effects for inflammatory neutrophils. The anti-inflammatory effects of Chol-HCQ liposomes also contributed to the anti-fibrotic abilities. In the pulmonary fibrosis rats induced by bleomycin, Chol-HCQ liposomes inhibited the phosphorylation of NF-κB and ERK1/2. The inhibition of ERK and NF-κB might contribute to the resolution of early inflammation in the pulmonary fibrosis development. There are reports demonstrating that inhibition of NF-κB and ERK signalling pathways resulted in the suppression of CTGF^32, 33^. We detected the expression of CTGF in rat lung tissues with bleomycin-induced pulmonary fibrosis. The results demonstrate that Chol-HCQ and HCQ liposomes inhibited CTGF expression.

NF-κB activation plays a central role in pro-inflammatory activities through its ability to induce the release of multiple pro-inflammatory cytokines^34^, and inhibiting TNF-α activities in many diseases has been remarkably successful in therapy^35^. We observed that Chol-HCQ inhibits the NF-κB activation *in vitro* and *in vivo*, and it suppressed the expression of TNF-α in the plasma from IPF rats and lung macrophages treated with bleomycin; the TNF-α inhibitor etanercept was used as control. Inhibition of NF-κB activity^36^ and suppression of TNF-α was reported as an anti-inflammatory therapy in bleomycin-induced pulmonary fibrosis^37^. Chol-HCQ liposomes dramatically eliminated inflammatory neutrophil infiltration in bleomycin-treated rats. These findings show that Chol-HCQ liposome administration is a potential strategy against pulmonary fibrosis inflammation and promotes anti-fibrosis activities. The inhibition of ERK and NF-κB might contribute to the resolution of early inflammation in the pulmonary fibrosis development, but more researches designs for the mechanism study are needed.

In summary, our study clearly demonstrated that Chol-HCQ inhibited the development of bleomycin-induced pulmonary fibrosis in rats by reducing regenerative fibroblast proliferation, suppressing inflammation and inhibiting the CTGF/ERK pathways. Taken together, these results provide *in vitro* and *in vivo* evidence that Chol-HCQ liposomes may have therapeutic potential for the treatment of pulmonary fibrosis induced by bleomycin. However, there are some limitations of this study. Bleomycin-induced pulmonary fibrosis was thought be an “inflammatory” model, thus other models such as silica or radiation-induced pulmonary fibrosis are need to test the anti-fibrosis effect of Chol-HCQ liposomes. For the limitations of the present study, further studies are needed to investigate alternative and additional mechanisms and the anti-fibrotic effects in other pulmonary fibrosis models, including radiation- and silica-induced models.

## Methods and Materials

### Animals

Female Sprague Dawley rats (aged 7–9 weeks, weighting 250–280 g) were purchased from Vital River (Beijing, China). The rats were housed and maintained under SPF conditions in a facility. All of the animal experiments were performed according to the guidelines of the Institutional Animal Care and Use Committee of Sichuan University(Chengdu, Sichuan, China) and protocols were approved by the Institutional Animal Care and Use Committee of Sichuan University (Chengdu, Sichuan, China).

### Establishment of bleomycin-induced pulmonary fibrosis in rats

The rats were administered a single intratracheal instillation of bleomycin sulphate dissolved in saline (5 mg/kg body weight; Melone Pharmaceutical Co., Ltd, Dalian, China) on day 0 of the experiment to induce pulmonary fibrosis as previously described^38^, while an equal volume of saline was injected into the rats from the control group.

### Synthesis and characterization of Chol-HCQ

The Chol-HCQ synthesis route is presented in Supplementary Figure 1. Chol-HCQ was synthesized through a simple two-step reaction. Briefly, cholesterol and succinic anhydride were used to prepare the intermediate Chol-suc, which was subsequently reacted with HCQ to form Chol-HCQ. The mixture containing cholesterol, succinic anhydride and dimethylaminopyridine (DMAP) (1:2.5:2.5, molar ratio) in dichloromethane reacted to generate the activated cholesterol ester. The reaction mixture was stirred at room temperature and monitored at the same time. Once cholesterol was no longer detected, the reaction was terminated. The reaction liquid was washed with 1 M HCl solution and then the dichloromethane was removed by rotary evaporation. The crude Chol-suc was washed by with ethanoic acid solvent and the white crystal product remained after the solvent was removed; the Chol-suc was characterized by ^1^H-NMR and ^13^C-NMR spectroscopy. Cho-suc, HCQ, DMAP and EDCI (1.2:1:1.2:1.2, molar ratio) were mixed in chloroform and the reaction mixture was stirred at room temperature and monitored at the same time. When the reaction was terminated, the product was separated by column chromatography. The mixture was loaded on the column after removal of most but not all of the chloroform and separated via gradient elution with dichloromethane and methanol. The product Chol-HCQ was characterized by ^1^H-NMR and^13^C-NMR. ^1^H and ^13^C-NMR spectroscopy was performed on a Varian spectrometer (Varian, Palo Alto, CA, USA) model Gemini 400 at 400 MHz. The purity was analysed by high performance liquid chromatography (HPLC).

### Liposome preparation and administration

HCQ and Chol-HCQ-loaded liposomes were prepared by a film dispersion method. Chol-HCQ and phosphatidylcholine (PC) (w/w 1:1) (Alabaster, AL, USA) were dissolved in chloroform. The solution was evaporated on a rotary evaporator to remove the organic solvent and then the film was hydrated in pH 7.4 phosphate buffer solution (PBS) at 60 °C. The suspension was sonicated and sterilized through a Millipore 0.22μm microporous membrane (Millipore Corporation, Billerica, MA, USA) and stored at 4 °C. The null liposome was prepared by the same method and composed of cholesterol (Shanghai Bio Life Science & Technology CO. Ltd) and PC (w/w 1:1). HCQ liposomes consist of three components, HCQ, PC and cholesterol (w/w 1:0.5:0.5). The liposome particle sizes range from 100-150 nm and the Zeta potentials were near zero. The liposomes mean particle size and Zeta potential were measured with a ZetasizerNanoZS ZEN3600.

Bleomycin-treated rats were randomly divided into four experimental groups and intravenously administered treatments daily. The doses of Chol-HCQ and HCQ liposomes were 20 mg/kg and 8 mg/kg, respectively, and PBS solution and PC liposomes were used as controls.

The Chol-HCQ and HCQ encapsulation efficiencies were determined by a simple method. Fresh liposomes were isolated from an aqueous suspension medium by ultracentrifugation at 20,000 rpm for 30 min. The supernatant, which might contain free drug, was tested by high performance liquid chromatography (HPLC), the lower quantification limit of which was 32.25 ng/ml (as described in the HPLC section). The concentrations of Chol-HCQ and HCQ were under this limitation or un-detectable. The drugs were thought to be totally incorporated into the liposomes, and we calculated the concentrations as the initially added drug.

### Histopathological examination and immunohistochemistry staining

Rats were sacrificed on days 7, 14 and 28 of the experiment. Lung tissue specimens were fixed in a 4% (m/v) PBS-buffered paraformaldehyde solution, dehydrated using graded ethanol and embedded in paraffin. Serial sections (5μm) were cut and stained with hematoxylin and eosin or Masson’s trichrome to evaluate the histopathological changes and the degree of accumulated collagen fibres. A Naphthol As-D Chloroacetate Kit (Sigma-Aldrich, St. Louis, MO, USA) was used for neutrophil esterase staining. Paraffin sections were permeated in 0.1% Triton X-100 (Sigma-Aldrich, St. Louis, MO, USA), incubated with anti-rat CTGF antibody (AbcamPLC, Boston, MA, USA) overnight at 4 °C, and then incubated with horseradish peroxidase (HRP)-conjugated secondary antibodies for immunohistochemistry staining.

### Lung fibroblast proliferation assay

Rats treated with bleomycin as described above were killed on day 14 and lung fibroblasts were isolated as described previously^39^. The cells were cultured in DMEM (Gibco, Invitrogen Corp., Carlsbad, CA, USA) containing 10% foetal bovine serum. Only early cells (passages 4–8) were used for the experiments in this study^40^. Lung fibroblasts were examined by α-tubulin immunofluorescent assay at passage 4. MTT assay and EdU immunofluorescent staining were conducted for lung fibroblast proliferation analysis. MTT assay was performed as described previously^41^. A 5-Ethynyl-2′-deoxyuridine (EdU) staining Kit (RiboBio, China) was used to detect the proliferating cells according to the manufacturer’s instructions.

### Apoptosis analysis

Lung fibroblasts were treated with graded concentrations of Chol-HCQ that were dissolved in DMSO (0, 5, 10, 20, 30, 40, 50, and 100 μM) for 48 hours and examined with a FACS Caliber Flow Cytometer (BD Biosciences, San Jose, CA, USA). Propidium iodide (PI) and Annexin V labelled with fluorescein isothiocyanate (FITC) were used to determine the cell viability and to assess the PS exposure, respectively, and were performed according to the manufacturers’ instructions (Keygen Biotech, China). The Annexin V-positive PI-negative cells represented the apoptotic cells^42^.

### Quantitation of BAL cells

Bronchoalveolar lavage (BAL) fluid was collected by lavaging the lung with 5 mL PBS via a tracheal catheter. The total number of BAL cells, BAL eosinophils, lymphocytes, neutrophils and macrophages were counted in Wright-Giemsa-stained cytospins.

### Isolation of alveolar macrophages and treatment with Chol-HCQ

As we have described before, BAL was performed by instilling 5 mL of serum free DMEM media. BAL fluid was incubated in 24-well plates for 2 hours at 37 °C and 5% CO_2_. The attached cells were analysed by flow cytometry, and more than 80% of the cells were positive for CD11b (BD Biosciences, San Jose, CA, USA) and F4/80 (BD Biosciences, San Jose, CA, USA). Isolated alveolar macrophages were stimulated with bleomycin (25 μg/ml) and treated with Chol-HCQ (10 μM) or HCQ (10 μM) and etanercept (5 μg/ml) in the presence of brefeldin A (GolgiPlug, BD Biosciences, San Jose, CA, USA) in a 24-well plate at 37 °C for 24 h. For macrophage intracellular cytokine detection, cells were stained with APC-CD45 antibody (BD Biosciences, 1:100), PerCP-Cy5.5-CD11b antibody (BD Biosciences, San Jose, CA, USA, 1:100) and FITC-F4/80 antibody (BD Biosciences, San Jose, CA, USA, 1:100) for 30 min in PBS at 4 °C and then washed twice with PBS. The cells were fixed in a 2% paraformaldehyde solution for 20 min, permeabilised using 1% Tritox-100 for 30 min at 4 °C and washed with PBS. Then, the cells were stained with PE-TNF-α antibody (BD Biosciences, San Jose, CA, USA, 1:100) for 2 h at 4 °C, washed, and examined with a FACS Caliber Flow Cytometer (BD Biosciences, San Jose, CA, USA).

### Whole blood Chol-HCQ and HCQ detection assay by HPLC

Chol-HCQ and HCQ concentrations in rat whole blood were assayed by using high performance liquid chromatography (HPLC). The HPLC system was composed of a Waters Alliance 2695 separation module (Empower software) and a Waters 2996 photodiode array detection system (190–800 nm). The column is an Atlantis dC_18_ column (4.6 × 150 mm, 5μm, Waters, Milford, USA). The mobile phase contained 60% methanol and 40% water in the isocratic elution mode and model within 20 min. The flow rates were set at 1 mL/min and the temperature was 50 °C. Regarding sample preparation, 10 μl of HCQ sulphate or Chol-HCQ (internal standard) at 1 mg/mL was added to 90 μl of whole blood. After vortex for 5 min, 500 μl of ethyl acetate was added before a 5-minute vortex step. Then, the tubes were centrifuged at 13000 rpm for 10 minutes at room temperature. The supernatant (500 μl) was transferred into a tube and dried under nitrogen plastic. Next, 20 μl ethyl acetate was added to the tubes followed by a 5-minute vortex step. Then, 80 μl methanol was mixed in the tubes, the tubes were centrifuged at 13000 rpm for 10 minutes at room temperature, and 80 μl of each sample was transferred into a plastic vial for chromatography. Calibration curves were linear from 31.25 to 5000 ng/ml. The intra- and inter-assay coefficients for the analytical variability were both less than 10%. The lower quantification limit was 32.25 ng/ml. For the whole blood sample treatment, 500 μl of each sample was treated as described for the HPLC test.

### Western blot analysis

The lung tissues from the experimental rats and lung fibroblasts treated with Chol-HCQ were homogenized in RIPA lysis buffer (Beyotime Biotech, China) containing 1 mM phenylmethylsuphonyl fluoride. The lysates were then centrifuged at 13,000 rpm for 15 min at 4 °C and the supernatants were collected and stored at −80 °C. A BCA protein assay kit (Pierce, Thermo Fisher Scientific, Inc., Waltham, MA, USA) was used to determine the protein concentrations. Equal amounts of protein were loaded and run on 10% SDS-PAGE gels, transferred onto Millipore PVDF membranes and blocked with 4% BSA. Then, the membranes were incubated with primary antibodies at 4 °C. The following primary antibodies were used: (1) anti-NF-κB (Santa Cruz Biotechnology, Inc., Santa Cruz, CA, USA), (2) anti-phospho-NF-κB (Santa Cruz Biotechnology, Inc., Santa Cruz, CA, USA), (3) anti-ERK1/2 (AbcamPLC, Boston, MA, USA), and (4) anti-phospho-ERK1/2 (Thr202/tyr204) (AbcamPLC, Boston, MA, USA). Antibodies were detected with horseradish peroxidase (HRP)-conjugated secondary antibody and the blots were developed with the ECL-Plus reagent (Millipore, MA, USA). The blots were tested for GAPDH (AbcamPLC, Boston, MA, USA) to confirm equal protein loading.

### ELISA analysis

The levels of anti-inflammatory markers in plasma, including TNF-α and TGF-β, from the experimental rats were measured using commercial ELISA Kits (eBioscience, San Diego, USA). All of the experimental procedures were performed according to the manufacturer’s instructions.

### Statistical analysis

Groups were compared with the Prism software (GraphPad) using a two-tailed unpaired Student’s *t*-test or Dunnett’s t-test. Data are presented as the mean ± s.e.m.

## Electronic supplementary material

Cholesterol-modified Hydroxychloroquine-loaded Nanocarriers in Bleomycin-induced Pulmonary Fibrosis
