# Supplementary material for: Cholesterol-modified Hydroxychloroquine-loaded Nanocarriers in Bleomycin-induced Pulmonary Fibrosis
Source: Sci Rep. 2017 Sep 6;7:10737. doi: 10.1038/s41598-017-11450-3 (PMC5587549; doi:10.1038/s41598-017-11450-3)
Supplement: Supplementary file 1 — Cholesterol-modified Hydroxychloroquine-loaded Nanocarriers in Bleomycin-induced Pulmonary Fibrosis [file 41598_2017_11450_MOESM1_ESM.pdf]

## Supplementary Information

# Cholesterol-modified Hydroxychloroquine-loaded Nanocarriers in Bleomycin-induced Pulmonary Fibrosis

Li Liu[a], Jun Ren[a], Zhiyao He, Ke Men, Ye Mao, Tinghong Ye, Hua Chen, Ling Li, Bocheng Xu, Yuquan Wei*, Xiawei Wei*

State Key Laboratory of Biotherapy/Collaborative Innovation Center, Sichuan University, No. 17, Block 3, Southern Renmin Road, Chengdu, Sichuan 610041, China

a These authors contributed equally to this work.

* Corresponding author

Correspondence: Yuquan Wei E-mail: yqwei@scu.edu.cn

Xiawei Wei E-mail: xiaweiwei@scu.edu.cn

Conflict of Interest Statement

The authors declare no conflict of interest in the subject of this manuscript.

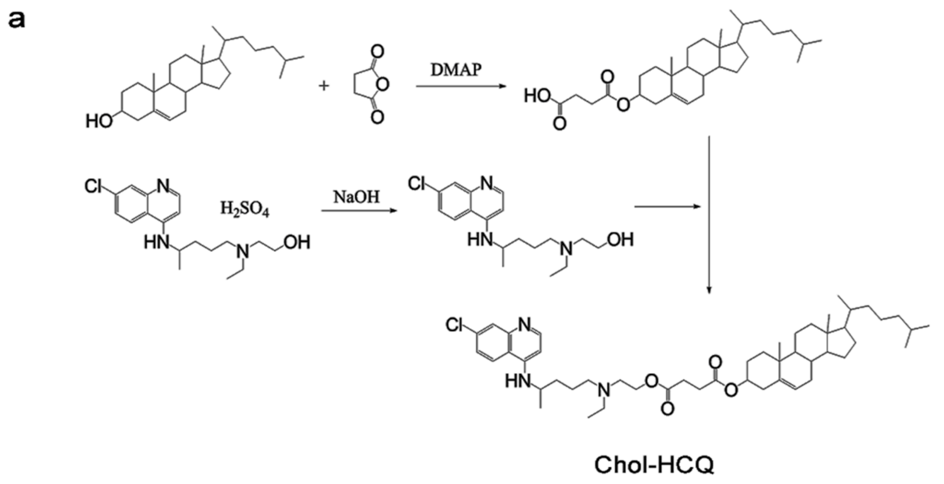


**Supplementary figure 1 The general procedure for the synthesis of Chol-HCQ.** (a) The Chol-HCQ synthesis route. As described in Methods, Chol-HCQ was synthesized through a simple two-step reaction. Specifically, cholesterol and succinic anhydride were used to prepare the intermediate Chol-suc, which was subsequently reacted with HCQ to form Chol-HCQ.

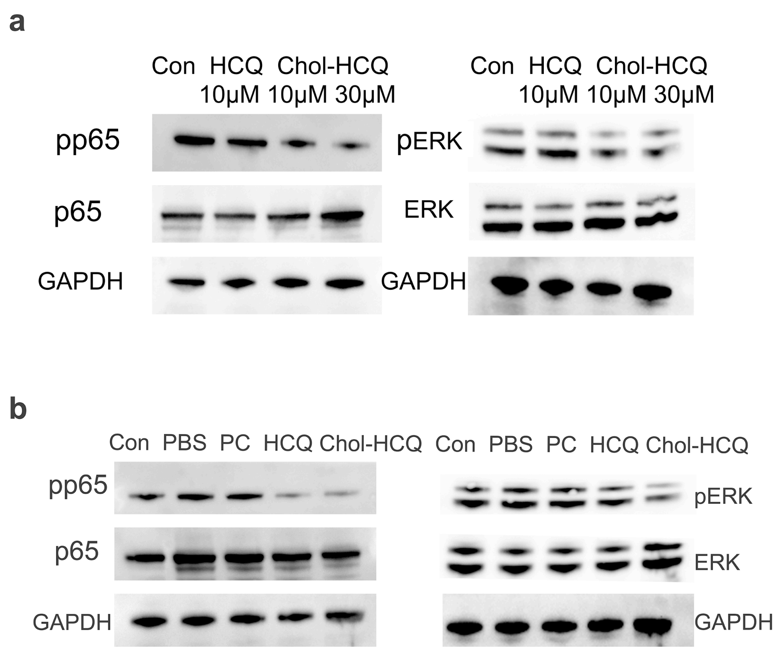


**Supplementary figure 2 Original western blots of phosphorylated ERK1/2 and NF-κB in lung fibroblasts and tissues.**

The lung fibroblasts (a) and lung tissues (b) of experimental rats treated with Chol-HCQ were homogenized in RIPA lysis buffer. The western blot gels have been run under the same experimental conditions. The phosphorylation of ERK1/2(Thr202/Tyr204) and NF-κB were determined and GAPDH was used as internal control. The western blots showed here are original images in the main figure (Figure2, 4).

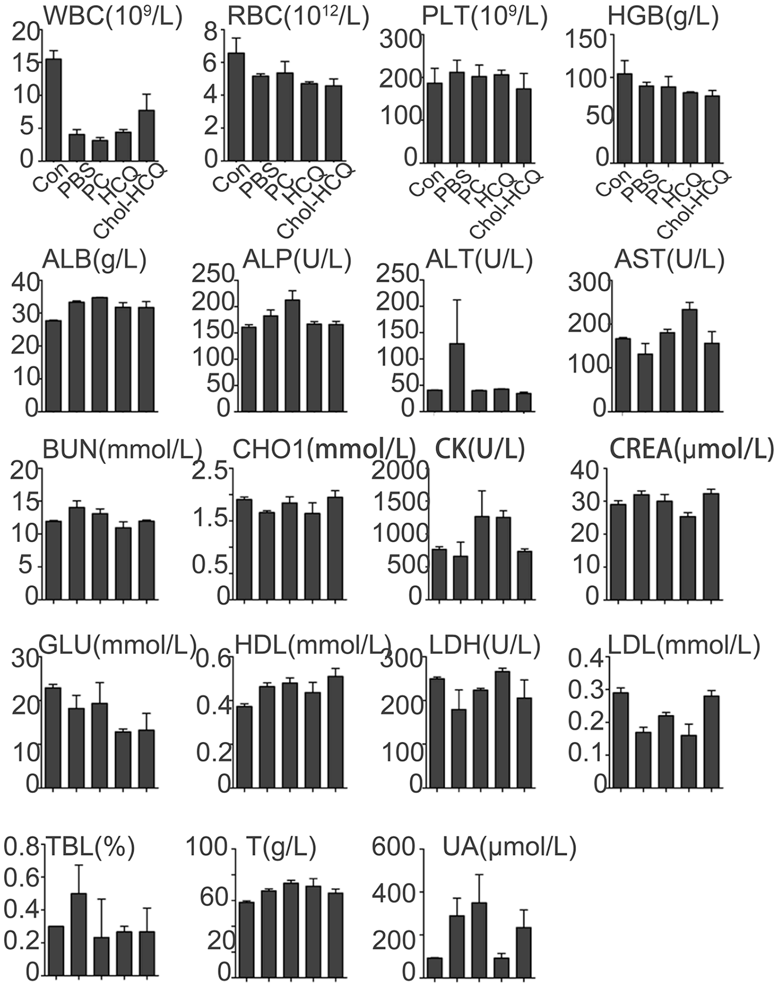


**Supplementary figure 3 Blood test and serological biochemical analysis for safety evaluation of Chol-HCQ liposomes.** To study the effects of Chol-HCQ liposomes on the physiology in experimental rats; blood test and serological biochemical analysis were performed on day 28. And all the biochemical indexes of Chol-HCQ liposomes or HCQ liposomes treated mice were approaching to the normal ranges. Data are representative of three separate experiments， n=6.
